# Role of the ketogenic diet in neurodevelopmental disorders: neuroprotection and clinical practice

**DOI:** 10.3389/fnut.2026.1936511

**Published:** 2026-09-18

**Authors:** Luqiang Sun, Liu Qu, Shuqing Gao, Juan Chen, Pan Wu, Haichuan Wang

**Affiliations:** 1 The Third Affiliated Hospital of Chengdu Medical College and Chengdu Pidu District People’s Hospital, Chengdu, China; 2 Sichuan Academy of Medical Science & Sichuan Provincial People’s Hospital, School of Medicine, University of Electronic Science and Technology of China, Chengdu, China

**Keywords:** attention deficit hyperactivity disorder, autism spectrum disorder, ketogenic diet, neurodevelopmental disorders, neuroprotection

## Abstract

Neurodevelopmental disorders (NDDs), such as autism spectrum disorder (ASD), occur early in life and affect cognitive and behavioral health and motor development. While hereditary factors contribute to the development of ASD, recent research has shown that environmental factors, notably nutritional factors, play important roles in determining the severity and expression of symptoms. The ketogenic diet (KD), a high-fat, low-carbohydrate dietary strategy, has well-established therapeutic significance, particularly in the management of epilepsy. Currently, the KD has recently gained popularity beyond therapeutic treatment, becoming popular as a general health and lifestyle option for healthy people. However, despite its long history, the effects of KD consumption on the development of NDDs are uncertain. This review highlights current literature on the effects of maternal KD on child brain development and NDD development, emphasizing the potential relevance of the KD in treating NDDs. We discuss the therapeutic implications of KD consumption and highlight the known effects of maternal KD on NDD development in children, potentially improving our understanding of NDD treatment. Assessments of the safety of KD and its long-term effects on the developing nervous system should be prioritized in future studies to help evaluate the feasibility, safety, and implementation of the KD in treating NDDs.

## Introduction

Neurodevelopmental disorders (NDDs) are complicated illnesses caused by both hereditary and environmental factors. In recent years, eating has been recognized as a possibly modifiable element that may help reduce neurodevelopmental symptoms, supporting normal brain development (1). Food balance, quality, and choice, as well as overall nutrient consumption, substantially affect brain structure and activity throughout life (2).

The ketogenic diet (KD) is composed of a high percentage of fats, a low amount of carbs, and appropriate amounts of protein and other nutrients. KD has been used for more than a century since its initial presentation in 1921 by Dr. Wilder at the Mayo Clinic (3). The KD, which was initially implemented for the treatment of epilepsy, especially refractory epilepsy (4), is now being used in the management of obesity (5), polycystic ovary syndrome (6), cancer (7), diabetes mellitus (8), traumatic brain injury (9), amyotrophic lateral sclerosis (10), mitochondrial myopathies (11), primary sarcopenia (12), and other pathological conditions (13). Recent research has shown that the KD could be a novel technique for treating NDDs; however, the underlying mechanism is still unknown. As a result, in this study, the effects of KD consumption on NDDs and the related mechanisms were analysed, presenting new approaches for preventing or treating NDDs.

## NDDs

NDDs are composed of a variety of categorized disorders and are defined by impaired behavioral, communicative, cognitive, and motor function that decrease brain function and hamper future development (14). The American Psychiatric Association (APA) initially made the Diagnostic and Statistical Manual of Mental Disorders, v.5 (DSM-5), available to the public in 2013, providing clinicians with a guidebook for symptom-based analysis and categorisation of NDDs from early childhood. All the factors are related to age at onset, severity, and functional limitations. The DSM-5 classifies the following disorders as NDDs: intellectual developmental disorders, autism spectrum disorder (ASD), attention deficit hyperactivity disorder (ADHD), specific learning disorders, and motor disorders. NDDs frequently co-occur with one another, a phenomenon called “homotypic co-occurrence” (15). Autism and ADHD, for example, are frequently identified in people who meet any of the five criteria, although other diseases, such as schizophrenia or bipolar disorder, do not meet the criteria even though their symptoms are comparable. NDDs often appear earlier in life, particularly during infancy and early childhood, when characteristic symptoms emerge. Epidemiological research has demonstrated that the prevalence of NDDs varies worldwide. For example, according to the US National Center for Health Statistics, in 2015, approximately 15% of children were diagnosed with a type of NDD. In Europe, the prevalence varied, with 0.48% of children in Southeast France and 3.13% in Ireland diagnosed with diseases associated with autism (16). The causes of NDDs, particularly the most common form, autism, are poorly known. Autism is complex, with 400–1000 genes associated with autism risk; nevertheless, the reported genetic variants account for only a small fraction of the disease burden (17, 18). Among environmental factors, nutrition appears to play an important role in the development and progression of autism. People on the autism spectrum frequently have different reactions to eating than individuals who are not on the spectrum do. Gastrointestinal (GI) difficulties, nutritional deficiencies (ND), binge eating, and a lack of diversity in diet are frequent factors that might affect general health and the severity of symptoms.

## The KD

Carbohydrate restriction is among the most effective nutritional treatments for restoring metabolic balance in patients on diets high in refined carbs. In this context, the KD has attracted increasing scientific interest as a low-carbohydrate, high-fat diet that can create a metabolic state characterized by nutritional ketosis (19, 20) (Figure 1). The KD is characterized primarily by significant carbohydrate restriction, generally less than 50 g/day. However, the therapeutic application of the KD is specifically characterized by a fat-to-nonfat ratio of approximately 3:1 or 4:1, which supports sustained ketone body synthesis and metabolic adaptation (21). This metabolic shift increases the circulating levels of acetoacetate, beta-hydroxybutyrate (beta-OHB), and acetone, together known as ketone bodies (22). After many days to weeks of persistent carbohydrate restriction, peripheral tissues and the central nervous system (CNS) adapt to ketone consumption as an alternative energy substrate, a process known as ketoadaptation (23).

**Figure 1:**
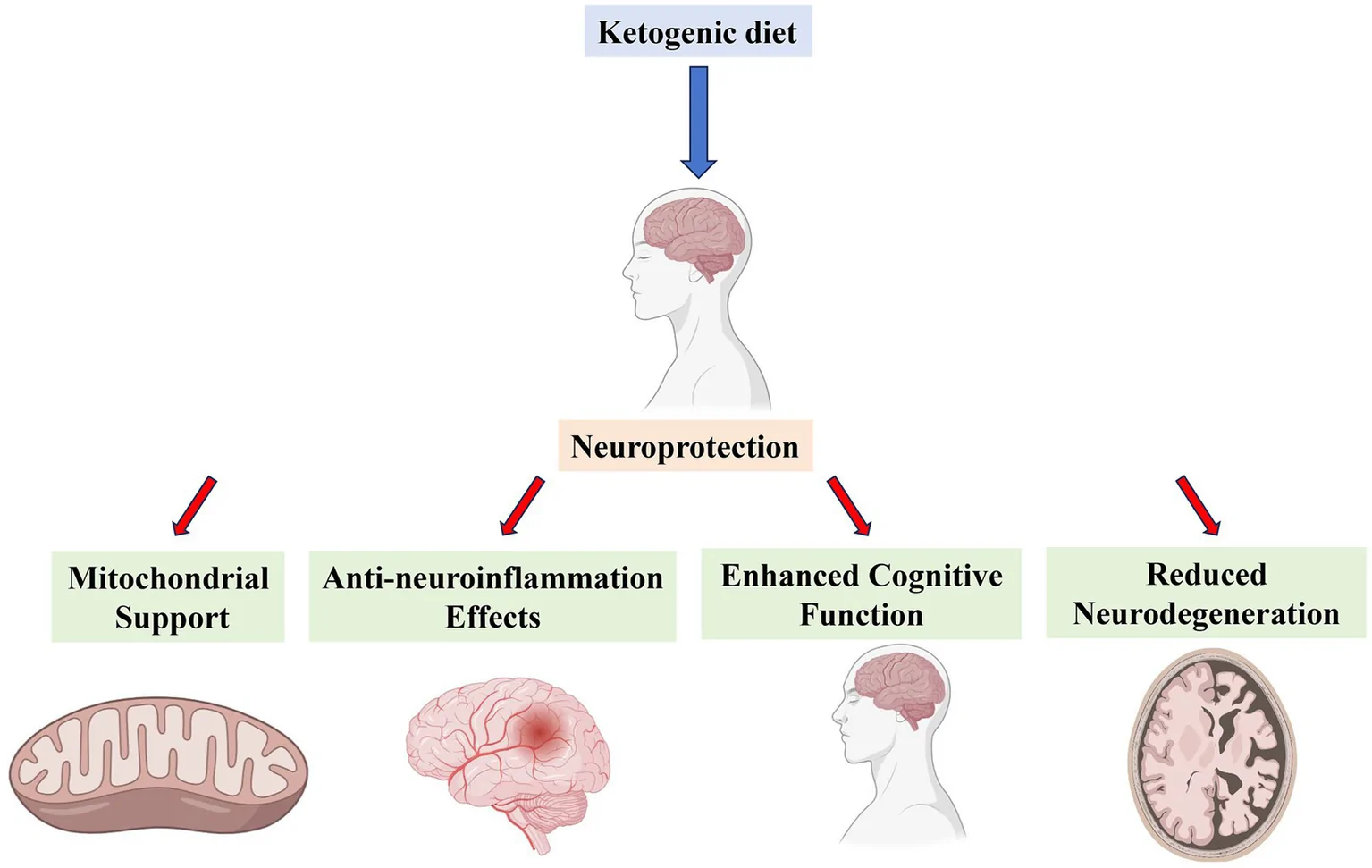
Proposed mechanisms underlying the relationship between ketogenic metabolism and neuroprotection in individuals with NDDs. KD consumption may affect energy metabolism, mitochondrial function, oxidative stress, and inflammatory signalling in the brain. Created with BioRender.com.

beta-OHB is a crucial ketone body for biological processes. It is an effective oxidative substrate for neural mitochondria, unlike acetone, which is mostly inhaled, and acetoacetate, which is quickly transformed (24, 25). In brain energy metabolism, ketone bodies bypass numerous glycolysis regulatory steps and enter the tricarboxylic acid cycle directly, potentially improving mitochondrial efficiency and reducing oxidative stress.

## Metabolism of ketone bodies by the CNS

Ketogenesis occurs mostly in the liver, specifically in the mitochondrial matrix of hepatocytes. However, this mechanism may occur in the kidneys and astrocytes to some extent (26). Astrocytes are the only cells in the CNS that can synthesize beta-OHB (27). The result of ketogenesis is the generation of ketone bodies. Free fatty acids (FFAs) from adipocytes are released into the bloodstream in response to increased levels of glucagon and epinephrine or decreased levels of insulin (28). FFAs undergo beta-oxidation, which produces acetyl coenzyme A (acetyl-CoA). Two acetyl-CoA units combine to generate acetoacetyl coenzyme A (acetoacetyl-CoA), which is then transformed into acetoacetate. Acetoacetate is the primary ketone and can break down into acetone or be reduced to create beta-OHB (28–30). As previously stated, beta-OHB is the main circulating ketone (31). Acetone, which accounts for the smallest proportion of ketone bodies, is expelled through the lungs (30). Owing to a shortage of enzymes for ketolysis, acetoacetate and beta-OHB are released into the circulatory system for metabolism in extrahepatic tissues. They diffuse across cell membranes, making them available for uptake by skeletal muscles and the brain. Monocarboxylate transporters (MCTs) on endothelial cells and astrocytes in the blood–brain barrier (BBB) transfer ketone bodies into the brain. MCT1 has a low affinity for ketone bodies and is found predominantly in the endotheliocytes and astrocytes of the BBB, whereas MCT2 has a high affinity for ketone bodies and is virtually exclusively expressed in the postsynaptic density of neurons (29). A percentage of FFAs in the bloodstream can penetrate the BBB and cause ketogenesis in astrocytes. Ketone bodies produced in astrocytes are subsequently transferred to adjacent neurons and used as energy substrates (Figure 2).

**Figure 2:**
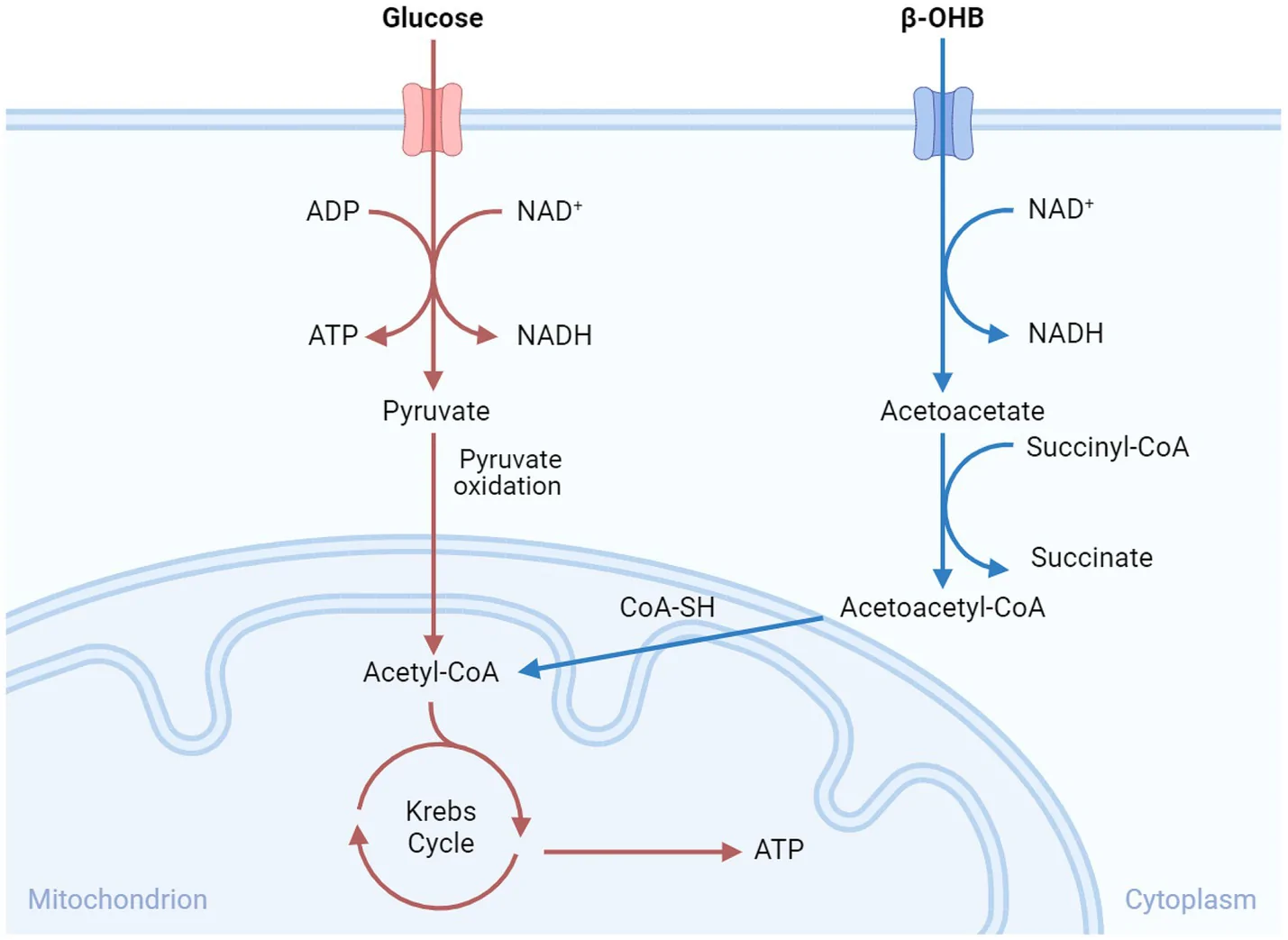
This diagram depicts the basic metabolism of beta-hydroxybutyrate in comparison to that of glucose and its contribution to the citric acid cycle in neurons.

According to previous reports, ketone bodies provide up to 70% of the energy needs of the rat brain during development (32); in the rat brain and striatum, beta-OHB levels peak at postnatal day seven and decrease significantly thereafter (33, 34). Neurons, astrocytes, oligodendrocytes, and microglia can metabolize ketone bodies, although their preferences for using them as energy or lipid sources differ. Many CNS cell types can use ketone bodies to replenish glucose and thus avoid the negative consequences of glucose deprivation. When glucose levels are low, neurons rely mostly on beta-OHB for energy (35). Astrocytes are the only brain cell type that can oxidize fatty acids and generate beta-OHB (36), and this energy is subsequently transferred to other types of brain cells. *In vitro* investigations have shown that prolonged beta-OHB treatment can protect primary astrocytes, hippocampal neurons, and C6 (rat glioma) cells from glucose deprivation (37, 38), and oligodendrocytes *in vitro* can use ketone bodies for cellular respiration (36). Similarly, the presence of beta-OHB during hypoglycaemia and glucose deprivation reduced reactive oxygen species generation both *in vivo* and in neural cells (39). Exposing stem cells, stem cell-derived neurons, primary neurons, and primary astrocytes to beta-OHB in the absence of glucose resulted in identical oxygen consumption, confirming the completeness of mitochondrial electron transport (40). Mesenchymal stem cells can develop into neuronal cells (41) and oxidize acetoacetate faster than glucose; additionally, acetoacetate oxidation generates fewer reactive oxygen species than glucose metabolism does (42). In glucose-deficient SH-SY5Y neuroblastoma cells, beta-OHB therapy prevents cell death by reducing reactive oxygen species levels and inhibiting ERK phosphorylation (43).

Additional research has focused on the effects of ketone body supplementation on glucose under cell culture conditions. An examination of rat and mouse neuroblastoma and glioblastoma cell lines revealed that ketone bodies are preferentially employed in lipid synthesis, whereas glucose remains their principal energy source (44). Additionally, high levels of beta-OHB inhibited glucose metabolism in guinea pig cortical brain slices (45) and reduced glucose intake when mixed in astrocyte cultures and hippocampal slices (46). However, beta-OHB increased the expression of glucose transporters in the mouse brain after fasting through epigenetic mechanisms, demonstrating a balanced interplay between metabolic control and glucose and beta-OHB levels (47). Ketone bodies influence microglial activity. Beta-OHB inhibits histone deacetylation, leading to enhanced microglial ramification (48), inhibited inflammasome activation (49), and decreased proinflammatory cytokine production following lipopolysaccharide exposure (50).

## Role of the KD in brain development

Brain development is a complex and dynamic process involving discrete stages that follow a specific order. The formation of a neural tube is the highlight of the embryonic stage. In the later stages, cortical development begins, followed by prosencephalon creation. Neuronal and glial proliferation subsequently occurs (51). Cell division, migration, network construction, maturation with differentiation, death of chosen cells, and myelination are all necessary processes for proper brain development (52). Such a complex process is energetically taxing and involves intensive transport of oxygen, protein, energy, and minerals from the mother to the foetus (51). During human pregnancy, the brain volume increases by up to 260% in the third trimester and more than doubles by the first year of life (51). The developing nervous system is susceptible to a variety of conditions. On the one hand, this is a positive trait because adequate stimuli cause more plasticity events, which are necessary for functional development. The “critical period” refers to the ability of the brain to restructure itself within specific developmental periods to adapt to its environment (52). Any imbalances or disruptions during important times of development can have long-term health effects on the progeny. Such phenomena are usually referred to as “developmental programming” (53). The maternal diet, particularly under- and overnutrition, affects offspring brain development (54). However, the effects of adherence to the KD during pregnancy or lactation on foetal brain development are not fully understood. According to Sussman et al. (55), alterations caused by the KD occur as early as the embryonic stage. These findings imply that in a mouse model, maternal KD consumption changes embryonic growth dynamics and region-specific development. The embryos of mothers who adhered to the KD were 37% larger than those of mothers who adhered to a standard diet (SD). However, as embryonic development progressed, their size decreased by 20%. The whole-brain volume of the embryos exposed to the KD, including the volumes of specific brain regions such as the midbrain, pons, and hypothalamus, decreased on E13.5 (embryonic day) but increased on E17.5. At E17.5, the size of the thalamus also increased. Similarly, the hearts of KD-treated animals were larger on E13.5 but were equivalent in size to those of the SD-treated animals on E17.5. These findings may indicate that maternal KD consumption delays foetal brain growth until later in pregnancy. KD-treated animals had considerably higher serum ketone and cholesterol levels. However, their serum triglyceride levels decreased, which contradicted the results reported in human subjects.

Other investigations have revealed similar decreases in both the whole-brain volume and the volumes of specific regions. Although not statistically significant, the brains of the KD-treated pups were slightly smaller on P11.5 (postnatal day). This trend continued until P21.5. Furthermore, larger parts of the cortex showed a relative loss in volume on the same postnatal day. The corpus callosum, fimbria, lateral ventricles, and hippocampus of those in the KD group were substantially smaller in size on P21.5. In contrast, the hypothalamus, spinal cord, and olfactory bulb areas were considerably larger in the KD group than in the SD group (56).

Another study by Sussman et al. (57) revealed significant region-specific alterations in brain volume associated with the KD, whereas overall, the brain volume remained unchanged. The volume of frontal cortex, cerebellum, and primary somatosensory cortex relatively increased, but the volume of hypothalamus, striatum, motor cortex, and auditory cortex relatively decreased.

Early-age adherence to the KD affects the metabolic profile of cortical tissue. Juvenile B6 and BTBR mice fed the KD had altered strain-specific metabolic pathways. Furthermore, methionine levels were decreased in BTBR mice. The level of carnitine decreased in the B6 mice fed the KD. KD consumption increased and decreased glutathione and inosine levels exclusively in the BTBR mice. In both strains, primary bile acid biosynthesis, one-carbon and folate metabolism, pantothenate and CoA biosynthesis, and amino sugar and nucleotide sugar metabolism were affected by KD consumption (58). Currently, even less is known about the influence of KD consumption on human brain development. Moreover, data concerning the long-term effects of maternal KD consumption on foetal development are insufficient.

## Role of the KD in NDDs

Previous studies have reported that adopting generally healthy diets during childhood, rather than focusing on particular nutrients, is connected to superior brain shape and cognitive ability later in life (59). Similarly, a review of randomized controlled studies by Gutierrez et al. revealed that adherence to balanced food patterns in general is also associated with superior cognitive results (60). In recent years, researchers have increasingly focused on whether the type of food consumed might impact the onset or progression of NDDs.

The KD has long been used to treat NDDs, particularly epilepsy. In a case study, Bujas-Petkovic et al. (61) connected KD consumption to symptom improvement in patients with Rett syndrome, which shares characteristics with autism. Recent studies have shown that KD consumption can decrease inflammation and positively alter the gut–brain axis (GBA) (62, 63). The modified keto diet, a more flexible version of the KD, is gaining popularity because it allows the consumption of more carbohydrates, which can be advantageous (63, 64).

Furthermore, the KD has received attention for its ability to reduce the core and related symptoms of ASD, mostly by inducing a metabolic shift from glucose metabolism to ketone body oxidation. This metabolic adaptation may increase mitochondrial efficiency, minimize oxidative stress, and restore the balance between excitatory and inhibitory neurotransmission, all of which are implicated in the pathophysiology of children with autism (65). For instance, BHB can reduce the activity of the vesicular glutamate transporter (VGLUT) in relation to KD-induced alterations in synaptic glutamate (66), thereby lowering the amount of glutamate in synaptic vesicles and possibly making it more accessible for the other functions mentioned above. Adenosine levels can increase in response to decreased glucose levels, and accessible adenosine can also increase in response to KD-induced reductions in adenosine kinase (ADK) (67). Both of these modifications increase the amount of extracellular adenosine in the synapse, thereby hyperpolarizing postsynaptic membrane potentials and reducing presynaptic glutamate release. Furthermore, as an essential neurotransmitter for neural excitability, glutamate has been deemed a mechanistic target of the KD, initially because of the consequences of altered metabolism in ketosis (68). Numerous studies have revealed the effect of KD consumption on ATP levels and mitochondrial activity. In mouse models, consumption of the traditional KD reduces the expression of genes linked to glycolysis and increases the mitochondrial load and expression of genes related to the tricarboxylic acid (TCA) cycle and oxidative phosphorylation in the hippocampus (23). Furthermore, a mutation in succinic semialdehyde dehydrogenase (SSADH), which disrupts GABA metabolism, can cause the KD to reverse the phenotype (69), likely through enhancing mitochondrial function. The KD reduces mitochondrial generation of ROS in the brain by increasing mitochondrial uncoupling (70) or through the transcription factor PPARγ2 (71), and other studies have shown that ketone bodies can have comparable effects (39).

Although the results are limited and are sometimes based on small samples, clinical and pilot studies have shown that people with ASD who adhere to the KD experience enhanced social interactions and have improved cognition and general behavior (72). Poor adherence, vitamin inadequacies, and a high reliance on animal-derived foods are all major issues that lead to health and environmental concerns. The KD modified by the addition of plant-based fat sources may represent a more balanced strategy, aiming to maintain the therapeutic advantages of the KD while lowering its ecological impact (73).

Compared with that in ASD, the role of the ketogenic diet in the management of ADHD is less well-established. Nonetheless, one of the most prevalent comorbidities linked to epilepsy is ADHD. Therefore, adherence to the KD as means to treat epilepsy may also benefit the management of ADHD symptoms. In a retrospective case study, two paediatric epileptic patients who adhered to the KD showed improvements in neuropsychiatric symptoms and seizure control. Approximately 60% of their symptoms were related to ADHD (74, 75). The study, which spanned more than 150 countries over a 28-year period, revealed a link between a high-fat diet—especially a plant-based diet—and a lower incidence of ADHD worldwide. Packer et al. fed dogs with both ADHD-like behavior and idiopathic epilepsy an MCT-enriched diet. The dog owners reported their dog’s behavior via a form. The canines showed signs of hyperactivity, excitability, and distractibility at baseline, according to the results. After a three-month nutritional intervention, the researchers noted significant improvement in the ADHD pursuing behavior and a decrease in stranger-directed fear (76). In two rat strains—Wistar and Long-Evans rats—fed a KD, exploratory behavior decreased and immobility increased. These results point to a potential improvement in the core symptoms of ADHD (77, 78). According to a recent study by Liu et al., the KD regulates the gut microbiota in SHR rats, normalizing symptoms associated with ADHD. Moreover, the expression of dopamine-related genes and the levels of the neurotransmitters noradrenaline and serotonin, as well as their second messengers, increased. Additionally, there was a noticeable change in the behavior of rats fed the KD; the overall distance travelled decreased and the immobility time increased in the SHR rats, indicating a reduction in the hyperactivity trait of ADHD (79).

These findings imply that adherence to certain diets, such as the KD, may improve outcomes for people with NDDs while simultaneously promoting increased environmental and health sustainability. The data presented here cover a wide range of study types, including clinical trials, systematic reviews, and analyses. Overall, these findings highlight the importance of sustainable dietary methods for enhancing both NDD management and overall ecological well-being.

## Safety of the KD as a therapeutic approach in clinical practice

The KD induces nutritional ketosis owing to persistent carbohydrate restriction, increased dietary fat consumption, and moderate protein intake. Although adherence to the KD may provide metabolic advantages in specific therapeutic circumstances, it is also linked to potential issues and may not be suited for all people (80).

Most medical professionals do not currently support adherence to the KD during pregnancy, possibly because of the lack of information pertaining to its safety for both the mother and the foetus. They instead stress the value of an individualized approach and close observation of the health of the mothers and the foetuses. Changes to the amount of fat and carbohydrates consumed should be made cautiously. For instance, flexible versions of the KD, with higher carbohydrate intake, are frequently advised to lower the risk of ketoacidosis during lactation (81).

Women who are pregnant or breastfeeding and adhere to the KD for therapeutic purposes should be monitored carefully for weight gain/loss, glycaemia, lipid biomarkers, and potential KD side effects. To minimize short-chain and polyunsaturated fatty acid deficiencies, the diet should include enough protein, good fats, and omega-3 s.

Individuals adhering to the KD should be under close medical supervision and should be monitored for maternal weight, glycemia, lipid markers, and nutritional parameters to ensure sufficient protein and omega-3 fatty acid intake, if clinically indicated (e.g., refractory epilepsy, epilepsy with childbearing potential, or glucose transporter type 1 deficiency syndrome) (81).

Furthermore, to mitigate the potential negative consequences of dysbiosis resulting from dietary changes, short-chain fatty acid supplementation could be considered and discussed with the patient. Current guidelines for pregnant women do not recommend the consumption of any additional short-chain fatty acids. However, in certain groups, such as pregnant, postpartum, and nursing women who have dysbiosis owing to an imbalanced diet, short-chain fatty acid supplementation may be beneficial. Probiotic supplementation increased overall short-chain fatty acid synthesis in malnourished children; however, the impact of supplementation with short-chain fatty acids, particularly acetate and propionate, was not statistically significant. However, probiotic administration led to a considerable increase in butyrate levels. Supplementation with omega-3 fatty acids and perhaps short-chain fatty acids should be used in conjunction with dietary modifications to improve maternal and foetal health.

## Conclusion

Here, we review current research on the potential role of the KD in NDDs. This review provides a more complete picture of how people with NDDs tolerate different food patterns over time. Ideally, studies should involve large groups of children with NDDs as well as neurotypical siblings from the same familial milieu to allow more precise comparisons. Furthermore, more emphasis should be placed on maternal diet during and after pregnancy, as well as the use of certain vitamins and natural medicines that may affect early gut and brain development.

Additionally, combining multi-omics methods such as genomics, gut microbiome analyses, proteomics, and metabolomics to gain a deeper understanding of the biological mechanisms underlying the link between diet and NDDs represents a new avenue for future research. This will help us find deeper mechanistic and causal linkages between nutrition and neurodevelopment, perhaps leading to more focused and individualized therapies in the future.

Since its inception, the KD has become more popular. Although it was initially implemented as a therapeutic approach to epilepsy, the KD is now a lifestyle and nutritional choice. However, the ramifications for nervous system development are not well known. The following aspects must be examined in future studies: standardized KD techniques, sex-specific analysis, and long-term human studies that incorporate microbiome, metabolite, and neurodevelopmental data.

Finally, it is critical to examine environmental impacts and sustainability issues. External variables such as air pollution, poor soil and water quality, and food production, processing, and transmission methods can affect the entire agrifood value chain, the nutrients ingested, and the balance of microorganisms in our bodies and environment. As a result, beginning early in life, adopting a more sustainable way of eating and living can help support both long-term health and optimal brain development.
